# Antimicrobial and Cytotoxic Assessment of Marine Cyanobacteria - *Synechocystis* and *Synechococcus*

**Published:** 2008-01-22

**Authors:** R. F. Martins, M. F. Ramos, L. Herfindal, J. A. Sousa, K. Skærven, V. M. Vasconcelos

**Affiliations:** 1 Departamento de Zoologia e Antropologia Dr. Augusto Nobre, Faculdade de Ciências, Praça Gomes Teixeira, 4099-002 Porto, Portugal; 2 Centro Interdisciplinar de Investigação Marinha e Ambiental, Rua dos Bragas 289, 4050-123 Porto, Portugal; 3 Escola Superior de Tecnologia da Saúde do Porto, Rua João de Oliveira Ramos, 874000-294 Porto, Portugal; 4 Department of Biomedicine, University of Bergen, Jonas Lies vei 91, N-5009 Bergen, Norway

**Keywords:** MarineCyanobacteria, Antibiotic, Cytotoxic, *Synechocystis*, *Synechococcus*

## Abstract

Aqueous extracts and organic solvent extracts of isolated marine cyanobacteria strains were tested for antimicrobial activity against a fungus, Gram-positive and Gram-negative bacteria and for cytotoxic activity against primary rat hepatocytes and HL-60 cells. Antimicrobial activity was based on the agar diffusion assay. Cytotoxic activity was measured by apoptotic cell death scored by cell surface evaluation and nuclear morphology. A high percentage of apoptotic cells were observed for HL-60 cells when treated with cyanobacterial organic extracts. Slight apoptotic effects were observed in primary rat hepatocytes when exposed to aqueous cyanobacterial extracts. Nine cyanobacteria strains were found to have antibiotic activity against two Gram-positive bacteria, *Clavibacter michiganensis* subsp*. insidiosum *and *Cellulomonas uda*. No inhibitory effects were found against the fungus *Candida albicans *and Gram-negative bacteria. Marine *Synechocystis* and *Synechococcus* extracts induce apoptosis in eukaryotic cells and cause inhibition of Gram-positive bacteria. The different activity in different extracts suggests different compounds with different polarities.

## Introduction

The potential contribution of marine organisms to the discovery of new bioactive molecules is increasingly challenging [1,2]. Natural products have been isolated from a wide variety of taxa and tested for various biological activities. Among these taxa, cyanobacteria are regarded as good candidates for drug discovery, with applications in agriculture [3], industry [4] and especially, in pharmaceuticals [5]. Although cyanobacteria are still primarily viewed as an environmental nuisance or a source of toxins, hazardous to man and aquatic livestock, there are many potential benefits to research on chemicals produced by these organisms. Antibacterial, antiviral, antifungal, algicide and cytotoxic activities have been reported [6-11]. The role of bioactive molecules in the producer organism itself is poorly understood but, considering the wide spectrum of biological adaptations and tolerance to environmental stress revealed by cyanobacteria, some of these compounds can be produced in an attempt to confer advantages for their survival. 

Research on biological active compounds produced by cyanobacteria has been focused especially on freshwater species. Studies on marine species are more restricted and are specially related to filamentous forms from tropical regions [10], Baltic Sea [12] and Mediterranean Sea [9]. In this work we describe biological activities of marine cyanobacteria strains belonging to the genera *Synechocystis* and *Synechococcus* isolated from the temperate Atlantic coast of Portugal. We screened marine cyanobacteria aqueous extracts and extracts obtained with organic solvents for antifungal and antibacterial activity. Cytotoxic activity was also investigated using primary rat hepatocytes and human HL-60 cells.

## Materials and Methods

### Cyanobacteria biomass

Fourteen marine cyanobacteria strains belonging to the genera *Synechocystis* and *Synechococcus* (Table 1), isolated from Portuguese rocky shores along the Northwestern, Central and South coastline were unialgal nonaxenic cultivated on a large scale for biological activity screening. 

**Table 1:** Marine cyanobacteria strains tested for antimicrobial activity

Strain designation	Genera	Strain	Genera
LEANCYA 1	*Synechocystis* sp.	LEANCYA 16	*Synechococcus* sp.
LEANCYA 5	*Synechocystis* sp.	LEANCYA 17	*Synechocystis* sp.
LEANCYA 7	*Synechococcus* sp.	LEANCYA 18	*Synechococcus* sp.
LEANCYA 10	*Synechococcus* sp.	LEANCYA 19	*Synechococcus *sp.
LEANCYA 11	*Synechococcus* sp.	LEANCYA 20	*Synechocystis* sp.
LEANCYA 13	*Synechocystis* sp	LEANCYA 21	*Synechocystis* sp.
LEANCYA 15	*Synechocystis* sp.	LEANCYA 22	*Synechococcus* sp.

Biomass was produced by cultivating the isolated strains in four litres of Z8 medium [13], supplemented with NaCl at a concentration of 20 gL^-1^. Cultures were maintained at 25 ºC, at a light intensity of 10 μmol m^-2^ s^-1^ provided by cool white fluorescent tubes and with a light/dark cycle of 14 h / 10 h. Cells were harvested after one-month of growth by centrifugation. Samples were frozen at -20 ºC, freeze-dried and then stored at -20 ºC.

### Extract preparation

For antimicrobial activity, extracts obtained with organic solvents and water extracts of the 14 strains were prepared at a concentration of 100 mg mL^-1^(freeze dried material/mL of solvent). Extractions were carried out successively with *n*-hexane, dichloromethane, methanol and water to extract compounds with increasing polarity [14]. Solutions were sonicated with an ultra-sound probe (Vibra Cell 50 – sonics & Materials Inc. Danbury, CT, USA) for 3x2 min on ice. The solutions were centrifuged at 10053 g for 10 minutes, the supernatants recovered and stored at -20 ºC. 

For cytotoxic activity only strains LEANCYA 5, 11, 13, 16, 17, 18, 19, 20 and 21 were screened. Freeze dried cyanobacteria (10 mg) were suspended in ice-cold water (0.4 mL) and homogenised 3 times with 20 s pulses with an IKA Ultra Turrax T5 at 20,000 rpm at 4ºC. The samples were left on ice for 1 h in the dark before centrifugation at 20,000 x g for 10 min. The supernatant was collected and the pellet was resuspended, mixed thoroughly and re-centrifuged twice with water (0.2 mL). The combined supernatants represent the aqueous extract. The remaining pellet was resuspended in ice-cold 70% methanol (0.4 mL), left on ice for 1 h, centrifuged and washed as described above. The result was the methanol extract. The sediment was extracted further with ice-cold 1:1 (v/v) methanol-dichloromethane (0.4 mL) for 12 h in the dark. The extract was centrifuged, the pellet washed twice and the supernatants combined. This constitutes the dichloromethane extract. All extracts were dried in a vacuum centrifuge and resuspended in water (100 µL, aqueous extract) or 25% aqueous dimethylsulfoxide (100 µL, methanol and dichloromethane extracts).

### Antimicrobial screening assay

The antibiotic activity of the strains was tested against the yeast Candida albicans and a wide range of Gram-positive and Gram-negative bacteria (Table 2). Some of the tested bacteria were isolated from the marine environment. The objective was to use species with ecological relevance, with the purpose of using the potential natural antibiotic compounds to control animal disease, such as fish used in marine aquaculture. The antibiotic activity test for all extracts of all strains was based on the agar diffusion assay [15]. To obtain fresh bacterial cultures, the marine bacteria were first cultivated on half strength marine agar (Pronadisa) and the freshwater bacteria on TSA (Tryptic Soy Agar- Merck) supplemented with NaCl to a final concentration of 1%. All strains were incubated at the appropriate temperature (Table 2) for 24 h. 

**Table 2:** Gram-positive and Gram-negative bacteria strains tested for antibacterial activity of marine cyanobacteria strains. For each strain growth temperature and origin is presented. (ATCC: American Type Collection Culture; DSM = DSMZ: Deutsche Sammlung von Mikroorganismen und Zellkulturen GmbH, Braunschweig, Germany; NCIMB: National Collections of Industrial and Marine Bacteria LTD)

Bacteria strains	Gram	Temperature (ºC)	Origin of strains
*Bacillus cereus*	*+*	22	Freshwater srimp
*Bacillus megaterium* DSM 32	+	30	Not specified
*Cellulomonas uda DSM 20107*	+	30	Compost
*Clavibacter michiganensis* subsp. *insidiosum*DSM 20157	+	30	Not specified
*Micrococcusluteus* ATCC 49732	*+*	37	Clinical isolate
*Thiobacillus thioparus DSM 505*	*+*	30	Not specified
*Staphylococcus epidermidis ATCC 49134*	*+*	37	Clinical isolate
*Staphylococcus aureus*	+	22	Freshwater srimp
*Streptococcus parauberis*	*+*	22	Marine fish
*Aeromonas hydrophila*	*-*	22	Freshwater fish
*Aeromonas salmonicida* subsp. *salmonicida*	-	22	Freshwater fish
*Enterobacter cloacae*	*-*	22	Freshwater srimp
*Escherichia coli *B	*-*	22	Not specified
*Halomonas aquamarina* NCMB 557	-	22	Seawater
*Halomonas pacifica *ATCC 27122	-	22	Seawater
*Photobacterium damselae *subsp.* piscicida*	-	22	Marine fish
*Proteus vulgaris*	*-*	22	Freshwater srimp
*Pseudomonas doudoroff* NCMB 1965	-	22	Sea water
*Vibrio compbelli* ATCC 25920	-	22	Sea water
*Vibrio harveyi*	*-*	22	Marine fish
*Vibrio natriegens *ATCC 14048	-	22	Salt marsh mud
*Vibrio parahemolyticus* ATCC 27969	-	22	Blue crab hemolymph
*Vibrio fluvialis *ATCC 33812	-	22	River water
*Vibrio tubiashii *ATCC 19106	-	22	Oyster
*Vibrio vulnificus* ATCC 27562	-	22	Human blood
*Yersinia ruckeri*	*-*	22	River sediment

Fresh colonies were suspended in saline solution and streaked onto the surface of Mueller-Hinton (Merck) agar plates, supplemented with NaCl, 1% final concentration. Three sterilized blank paper disks (Ø 6 mm) impregnated with each extract to test were placed on the surface of the inoculated medium. Plates were incubated for 24 h at the correspondent appropriate growth temperature. As controls, three sterilized blank paper disks (Ø 6 mm) impregnated only with the solvents used for the extractions were tested for each microorganism. In all the cases after impregnation with the extracts and the solvents, the disks were kept overnight in a laminar flow bench first sterilized by UV, to evaporate the solvents. For sensitivity control of agar plates, standard antibiotic disks (Oxoid) Streptomycin (10 µg), Oxytetracycline (30 µg), Flumequin (30 µg), Ampicillin (10 µg) and Erythromycin (15 µg) were assayed for reference purposes. After incubation, each plate was examined and the diameter of the zones with complete inhibition of growth, including the diameter of the paper disk, were measure to the nearest millimetre using a ruler, and expressed in mm.

### Cytotoxic screening assay

Cyanobacterial extracts were tested against primary rat hepatocytes and HL - 60 cells. Primary rat hepatocytes were isolated from male Wistar rats (80-150g) as described by Mellgren *et al*. [16]. After filtration and washing, the hepatocytes were resuspended (4.0 x 10^5^ cells mL^-1^) in pre-gassed (5% CO_2_/95% O_2_) low-phosphate buffer (120 mM NaCl, 5.3 mM KCl, 0.01 mM KH_2_PO_4_, 1.2 mM MgSO_4_, 1.0 mM CaCl_2_, 10 mM Hepes (pH 7.4) supplemented with lactate (5 mM) and pyruvate (5 mM).

The HL-60 human monocytic leukaemia cells (ATCC no.: CCL-240) were cultured as suspension cells (150,000 and 300,000 cells ml^-1^) at 37ℜ?C in a 7% CO_2_ atmosphere in RPMI medium enriched with 10% heat inactivated foetal calf serum. 

For cytotoxicity screening, cell suspension was added to a 4% cyanobacterial extract and incubated for 1 hour (primary rat hepatocytes) or 18 h (HL-60 cells) in tissue culture plates (Nunc, Denmark). The experiments were stopped by adding buffered formaldehyde (2% formaldehyde in phosphate buffered saline (PBS), pH 7.4 with 0.5 μg mL^-1^ of the Hoechst 33342 DNA-stain). 

Apoptotic cell death was scored by differential interference microscopy for cell surface evaluation combined with studies of nuclear morphology. Cell shrinkage, surface membrane budding, and chromatin hypercondensation were decisive factors for apoptosis, whereas cell swelling and cell membrane leakage (inability to exclude trypan blue) were signs on necrosis. DMSO had no significant effect on hepatocytes, whereas it was mildly toxic to HL-60 cells (less than 8% apoptotic cells at 1% DMSO for 18 h). 

## Results and Discussion

**Figure 1:**
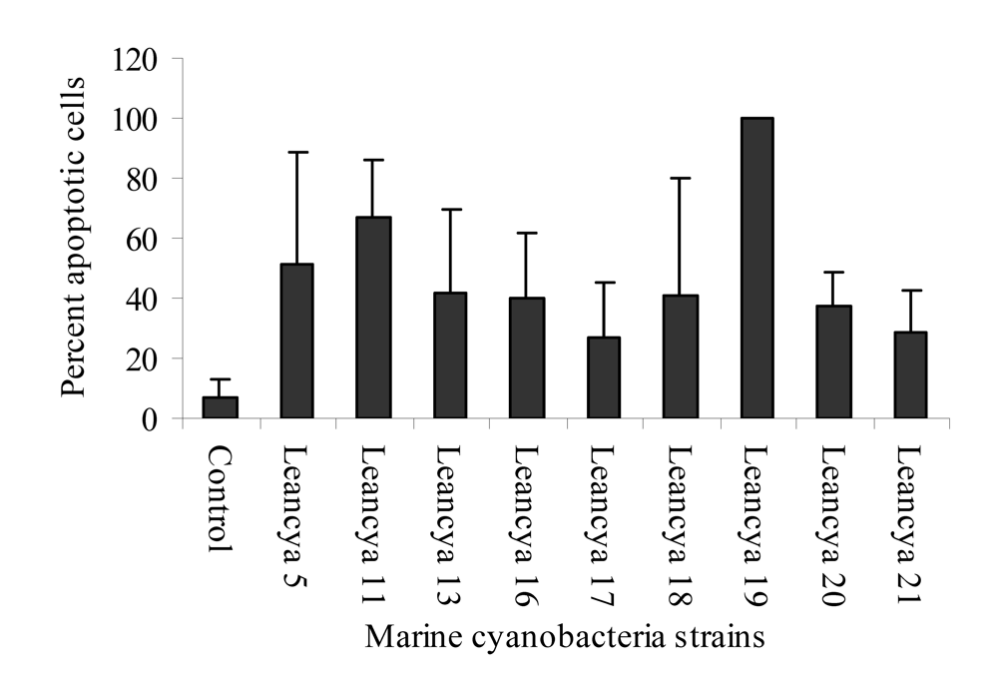
Percent apoptotic HL-60 cells following treatment with 0.4% marine cyanobacteria methanol extract. Results are the mean of triplicate experiments. Bars represent the standard deviation.

**Figure 2:**
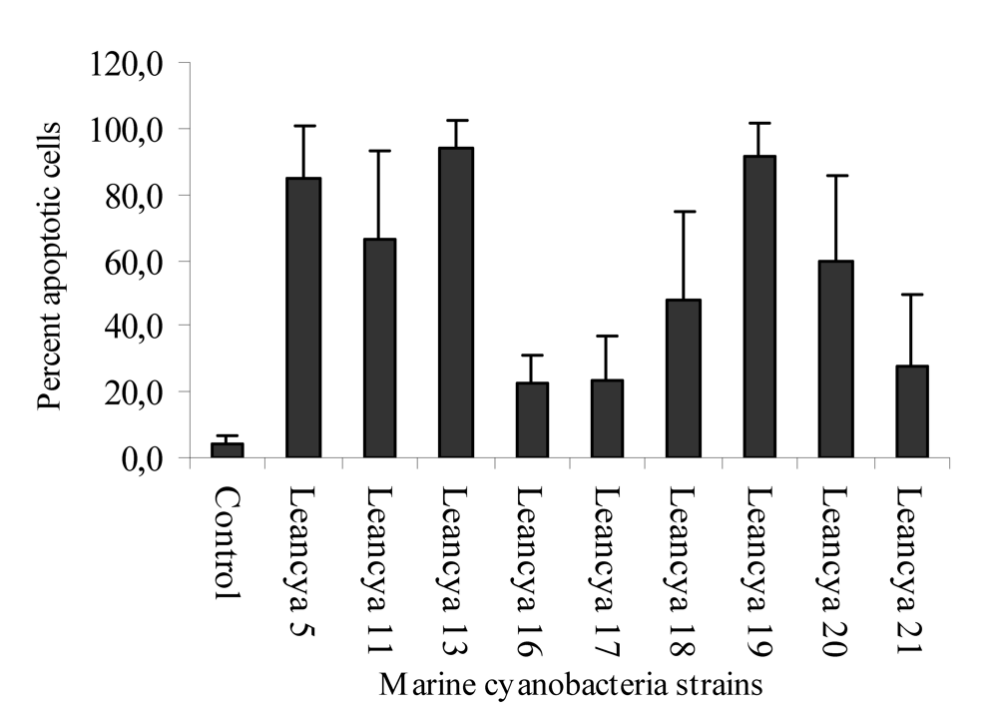
Percent apoptotic HL-60 cells following treatment with 0.4% marine cyanobacteria dichlormethane extract. Apoptosis results are the mean of triplicate experiments. Bars represent the standard deviation.

**Figure 3:**
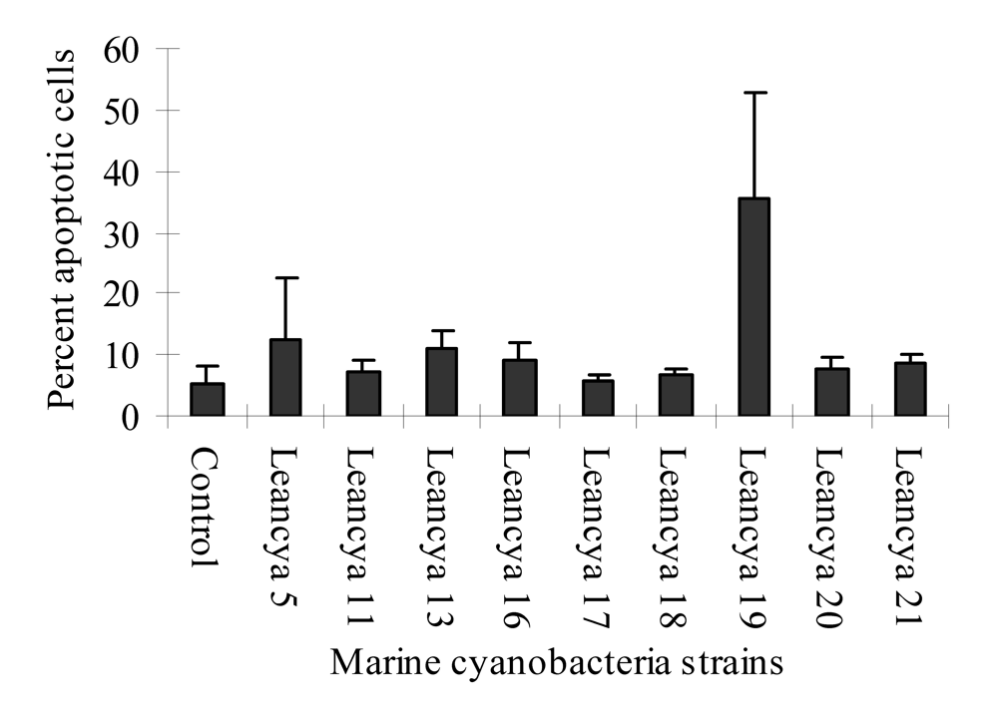
Percent apoptotic HL-60 cells following treatment with 0.4% marine cyanobacteria aqueous extract. Results are the mean of triplicate experiments. Bars represent the standard deviation.

For the cytotoxic assay using HL-60 cells, strong apoptotic effects were observed when cells were exposed to the methanol and/or dichloromethane extracts (Figure 1 and Figure 2 respectively). This was specially evident for strains LEANCYA 5, 11, 13, 19 and 20. In the aqueous extracts the most notorious effect was observed for strain LEANCYA 19 (Figure 3). The HL-60 cell death resembled apoptosis in most cases, but had clearly necrotic features with some extracts. However, this is probably secondary necrosis, since early (4 to 10 h) evaluation of the cells indicated apoptosis as seen by cell shrinkage and membrane budding (not shown). 

For the cytotoxic assays using primary rat hepatocytes, only the aqueous extract of the LEANCYA 11 strain showed mild toxicity (11.4% apoptotic cells). No significant apoptotic cells were registered for the treatments with the methanol and the dichloromethane extracts in any of the cyanobacterial strains tested.

With regards to the antimicrobial assays, the antibacterial effects of extracts from nine cyanobacteria strains against two Gram-positive bacteria, *Clavibacter michiganensis* subsp. *insidiosum* and *Cellulomonas uda* were determined. None of the tested extracts of the different cyanobacterial strains showed inhibitory effect on Gram-negative bacteria. In addition, no antifungal activity was found against the yeast *Candida albicans*. Table 3 summarizes all extracts that showed activity against the two gram-positive bacteria. Although the intensity of the inhibitory effects was variable between the extracts, methanolic extracts were the least toxic and the dichloromethane extract was the one with more pronounced inhibitory effects. In plates where disks were treated only with the solvents, no inhibitory growth was registered. The antibacterial substances used as control showed growth inhibition.

**Table 3:** Activity of extracts of marine cyanobacterial strains against two Gram-positive bacteria. Concentrations were of 100 mg/ml. Inhibition zones are measured in mm diameter including the diameter of the paper disk. (- No inhibitory effect; + < 10 mm; ++ < 10-15 mm; +++ < 15-20 mm)

Cyanobacteria strain	Extract	Gram-positive bacteria	
		*Clavibacter michiganensis* subsp. *insidiosum*	*Cellulomonas uda*
LEANCYA 5	Water	-	++
LEANCYA 7	Dichloromethane	+++	-
LEANCYA 10	Methanol	-	+++
	Water	+++	++
LEANCYA 11	Methanol	+++	++
LEANCYA 13	Hexane	++	++
	Dichloromethane	++	++
	Methanol	++	-
	Water	++	++
LEANCYA 16	Dichloromethane	+++	++
LEANCYA 19	Hexane	+++	+++
	Dichloromethane	++	++
	Methanol	++	++
	Water	-	+++
LEANCYA 20	Hexane	+++	-
	Dichloromethane	+++	-
	Water	+++	-
LEANCYA 22	Hexane	-	+++
	Dichloromethane	-	++

Marine cyanobacteria are known for the ability to produce cytotoxic compounds [17-19]. None of the tested extracts had microcystins. One of the cytotoxic effects of cyanobacteria is manifested by the capability to induce apoptosis in several eukaryotic cell lineages. Cyanobacterial extracts or purified compounds were found to cause apoptosis in hepatocytes, lymphocytes, endothelial cells, human skin fibroblasts, human embryonic intestine cells and promyelocytes HL-60 cells [7,20-22]. 

HL-60 cells are known to provide a useful model system to explore the ability of various substances to cause apoptosis [23]. These cells are an established model in biochemistry, as one can observe fundamental and critical signals involved in the activation of the body's immune system because of their ability to respond to foreign organisms. The cyanobacterial extracts tested in the present work against HL-60 cells clearly undergo apoptosis, as evidenced by DNA condensation. For this cell lineage the percentage of apoptotic cells was evident especially in the extracts obtained with organic solvents and, among these extracts, effects were particularly accentuated in the dichloromethane extract. 

Rat hepatocytes have been extensively used to investigate the induction of apoptosis by cyanobacterial secondary metabolites, specially due to the potential production of liver specific toxins such as microcystins and nodularins. Our results showed only slight toxic effects on primary rat hepatocytes which led us to exclude the production of these liver specific toxins or its production in small quantities. It is possible that the compounds that caused apoptosis in HL-60 cells can also cause apoptosis in hepatocytes but in a slower way and so more time is needed for observing the apoptotic phenomenon. One possibility is that the toxins act on mechanisms that are needed for cell division. HL-60 cells have a short cell cycle and compounds interfering with mitosis will lead to apoptosis within 4 to 10 hours. Such compounds have been found in cyanobacteria [24,25].

Antimicrobial activity of cyanobacteria refer almost to filamentous strains belonging to a wide range of genera [6,8-10,14,26]. Rao [6], working on the production of antibiotic compounds by filamentous forms of cyanobacteria, referred that the ability to produce antibiotic compounds might prevent bacterial contamination of the filaments and consequently pathogenic bacterial colonization. In contrast with research on antimicrobial compounds produced by filamentous cyanobacteria, relatively little has been reported on the antimicrobial activity of cyanobacteria of the Chroococcales group, namely of the genera *Synechoccystis* and *Synechoccocus*. As marine cyanobacteria tested in this research belong to *Synechoccystis* and *Synechoccocus* genera, our results emphasise the potential of these genera as a source of antibiotic compounds. In terms of antibacterial activity our results fits into the picture that antibacterial activity of cyanobacteria is primarily directed against Gram-positive bacteria [9,14]. This finding can be related to the fact that most Gram-negative bacteria are resistant to toxic agents in the environment due to the barrier of lipopolysaccharides of their outer membrane [27]. The difference in toxicity against Gram-positive and Gram-negative bacteria can also indicate that the mechanism of toxicity involves different functions in the two types of cells namely different permeability to the cyanobacteria compounds. In spite of the non toxic effect of cyanobacterial extracts to Gram-negative bacteria, we can have the possibility of the compounds to change the permeability of the cell outer membrane. This alteration could enhance the uptake of antibiotics, specially the hydrophobic ones, as it was demonstrated for the cyanobacterial toxin microcystin-RR [27]. 

None of the marine bacteria used as target organisms was inhibited by cyanobacteria extracts. In spite of these results we can not disregard the possibility of production of antibiotic compounds against marine bacteria since, with the exception of the specie *Streptococcus parauberis* isolated from marine fish (Table 2), all the marine bacteria tested were Gram-negative. Antifungal activities of cyanobacteria have been less found than antibacterial activities. Moore et al. [28] found that in more than 1000 cyanobacteria strains only 9% were able to inhibit fungal growth. Also Kreitlow *et al*. [14] found no activity against a fungi but inhibitory activity against Gram-positive bacteria. 

It is interesting to note that the cyanobacterial strains and extracts that caused higher percentage of apoptotic HL-60 cells were the ones that exhibited antibacterial activities. This is particularly true for strains LEANCYA 5, 11, 13, 19 and 20. It is possible that the same compounds can affect both eukaryotic and prokaryotic cell lines but it is also possible that the same cyanobacteria strain produces different bioactive substances targeted against different organisms or biochemical processes. 

In this work we were able to show that marine cyanobacterial strains of the genera *Synechocystis* and *Synechococcus* produce substances with inhibitory effects on prokaryotic cells and with apoptotic activity in eukaryote cell lines, which highlights the importance of these organisms as potentials pharmacological agents. Since different activities were observed in extracts obtained with organic solvents and extracts obtained with water we can suggest that compounds with different polarities are involved.
